# Data on crystal organization in the structure of the Fab fragment from the NIST reference antibody, RM 8671

**DOI:** 10.1016/j.dib.2017.11.013

**Published:** 2017-11-08

**Authors:** D.T. Gallagher, I. Karageorgos, J.W. Hudgens, C.V. Galvin

**Affiliations:** a Biomolecular Measurement Division, National Institute of Standards & Technology, Rockville, MD 20850, United States; b Institute for Bioscience and Biotechnology Research, 9600 Gudelsky Drive, Rockville, MD 20850, United States; c New College of Florida, Sarasota, FL 34243, United States

**Keywords:** Antibody, NIST, Pseudosymmetry, Standard, Twinning

## Abstract

The reported data describe the crystallization, crystal packing, structure determination and twinning of the unliganded Fab (antigen-binding fragment) from the NISTmAb (standard reference material 8671). The raw atomic coordinates are available as Protein Data Bank structure 5K8A and biological aspects are described in the article, (Karageorgos et al., 2017) [1]. Crystal data show that the packing is unique, and show the basis for the crystal's twinned growth. Twinning is a common and often serious problem in protein structure determination by x-ray crystallography [2]. In the present case the twinning is due to a small deviation (about 0.3 nm) from 4-fold symmetry in the primary intermolecular interface. The deviation produces pseudosymmetry, generating slightly different conformations of the protein, and alternating strong and weak forms of key packing interfaces throughout the lattice.

**Specifications Table**

**Table:** 

Subject area	*Biology, Molecular Biology*
More specific subject area	*Protein crystallography, Structural immunology*
Type of data	*Tables, Molecular graphics figures, Structural measurements*
How data was acquired	*Crystallography and molecular structure measurement*
Data format	*Analyzed*
Experimental factors	*Water was removed from the PDB file. Contacts are based on PDBePISA.*
Experimental features	*Packing geometry was analyzed to determine cause of crystal twinning*
Data source location	*Crystal diffraction was measured at the Advanced Photon Sources (aps.anl.gov). All other protocols and analysis were completed at NIST/IBBR*
Data accessibility	*Structural data is at*www.rcsb.org/pdb/explore.do?structureId = 5K8A

**Value of the data**

• These data describe the molecular packing in the atomic structure of a reference antibody Fab fragment, with implications for crystal growth and structure determination.
• The structure includes the complicating features of twinning and pseudosymmetry; these are described graphically and measured.
• The data provide evidence for the structural basis of the observed twinning.
• Although the described structure and packing are unique, twinning is a common problem in protein structure determination, and the described method/data provide an example that applies broadly to a large class of protein crystal structures.

## 1 Data

**Fig. 1:**
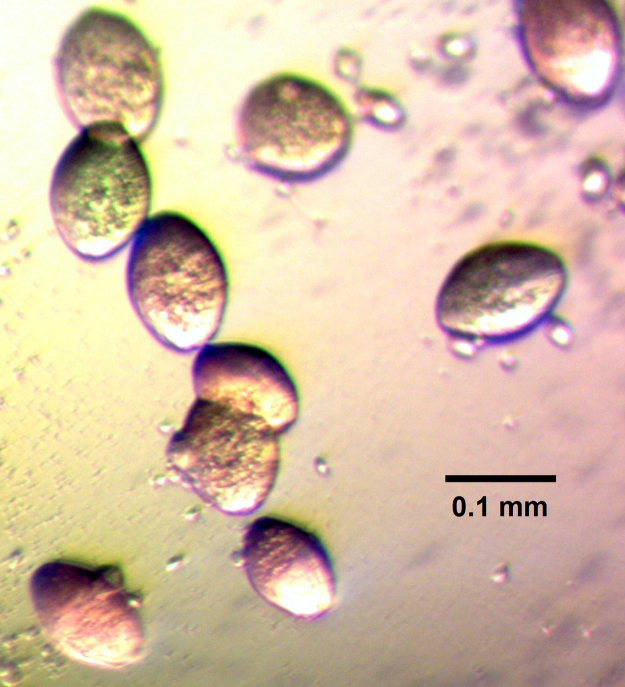
Football-shaped crystals of the NIST Fab (crystal form 2).

**Fig. 2:**
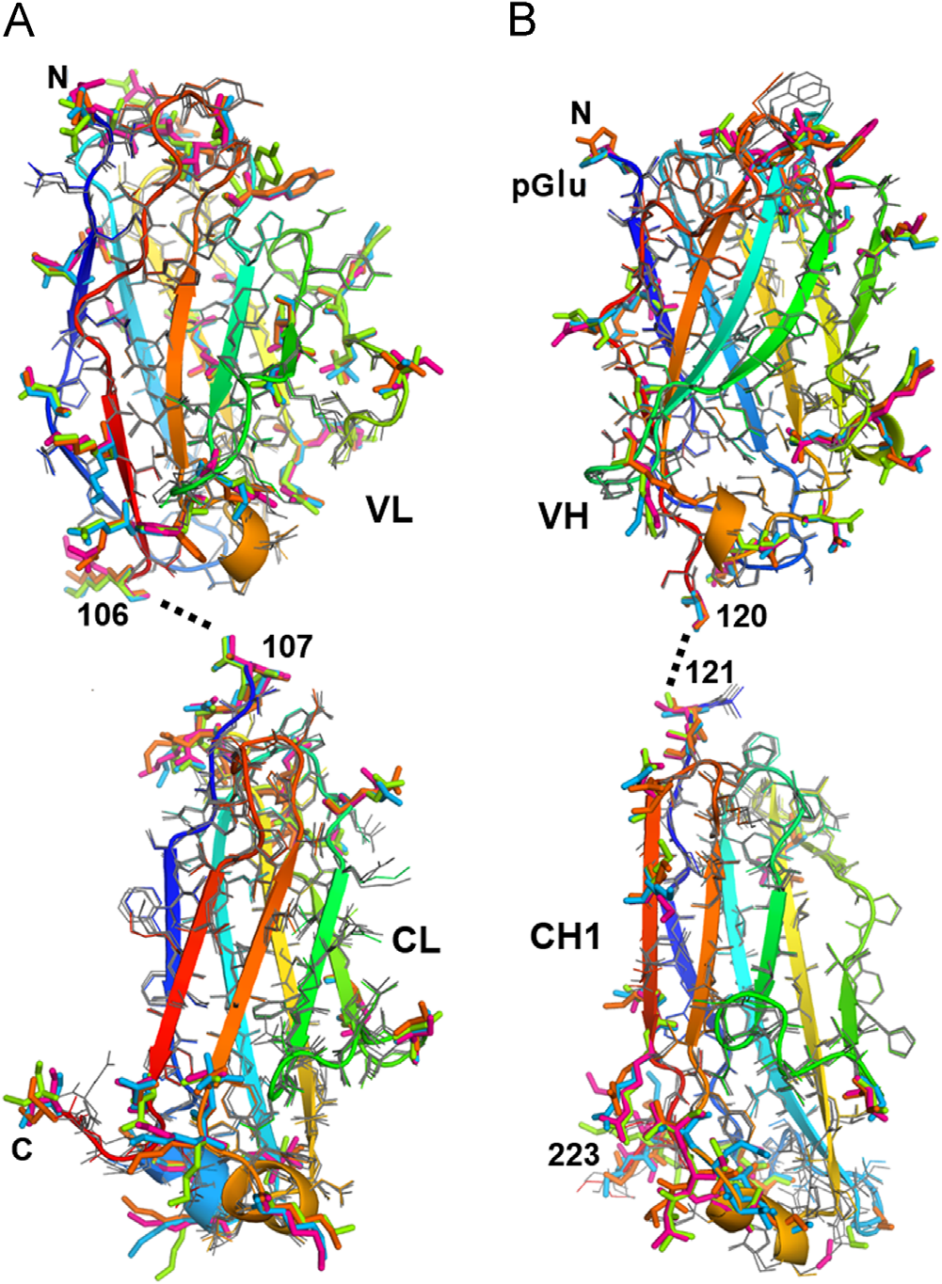
Superposition of the 4 independent Fabs in PDB structure 5K8A. For each folding unit of each polypeptide, its 4 independent structures are superposed. Backbone ribbons for L and H chains are shown using the rainbow color convention from blue to red, with the endpoints of each labeled. Sidechains with non-uniform rotamers are emphasized by thick bonds in varied colors; about 20% of the sidechains in each domain exhibit this rotamer variation, usually in surface loops that are involved in crystal contacts (contacts not shown). Residues with conserved rotamers are shown using thin bonds. Dashed lines show the breakpoints (at Fab elbows) used for the domainwise alignment. Note that the CDR loops are at the top in both chains. (A) Superpositions of the two folding units of the light chains (i.e., chains L, M, A and E). (B) Superpositions of the two folding units of the heavy chain (chains H, V, B, and F). The pyroglutamate residue (pGlu) at the N-terminus of the heavy chain is labeled.

**Fig. 3:**
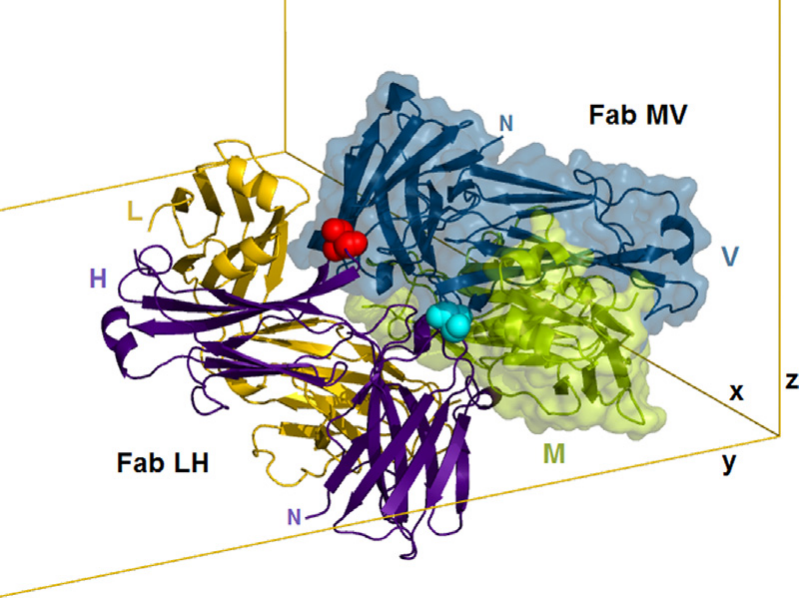
Lower half of the asymmetric unit, with 2 Fabs in close contact and related by a local dyad that is oblique to the cell. This contact, centered on the elbows of the 2 heavy chains (H and V), is highlighted by the dyad-related red and cyan residues. The interfaces that form this Fab dimer are listed in Table 2, rows Id=4 and Id=5. This 'dimer of Fabs' also occurs in a few differently-packed crystals of similar Fabs (e.g., 4JLR, 4MAU, 3L7F).

Table 1 gives primary crystal data, Fig. 1 shows the crystals, and Fig. 2 shows the structural variation in the four unique molecules in protein data bank (PDB) structure 5K8A [1], which is the 50 kDa Fab fragment derived from the NISTmAb standard antibody [3]. Table 2, calculated by the protein interface resource http://www.ebi.ac.uk/pdbe/pisa [4], and Fig. 3 thru 5 describe the crystal packing. Fig. 2 thru 4 show that the asymmetric unit (AU) contains 4 Fabs with slightly different conformations, and that there is an approximate dyad thru the center, so that although the space group is I222, the cell appears tetragonal and the crystal symmetry is pseudo-I422. The crystal packing geometry and pseudosymmetry are described using molecular graphics images.

**Table 1:** Primary crystallographic data for apo NISTmAb Fab structure (PDB:5k8a).

Diffraction	
Space group	I222
* a*, *b*, *c* (nm)	14.916, 14.920, 19.503
Resolution range (nm)	3.000 – 0.200 (0.205 – 0.200)
* R*_merge_^a^	0.083 (0.817)
Resolution (nm) at which <*I*/σ(*I*)> = 2	0.213
Completeness (%)	99.7 (97.6)
Redundancy	5.9 (4.5)
Refinement	
Nonhydrogen protein atoms	13,436
Water molecules	372
* R*_work_/*R*_free_	0.166/0.239
Overall Mean *B*-value (nm^2^)	0.449
Bond lengths rmsd from ideal (nm)	0.0008
Bond angles rmsd from ideal (degrees)	1.35
Residues refined	1742 (4 independent copies of Fab)

Data collection statistics for the highest resolution shell are given in parentheses.

a *R*merge = Σ |*I*-<*I*>| / Σ *I* where <*I*> is the mean of symmetry-related reflection intensities.

**Table 2:** Protein-protein contact interfaces in crystal 5K8A.

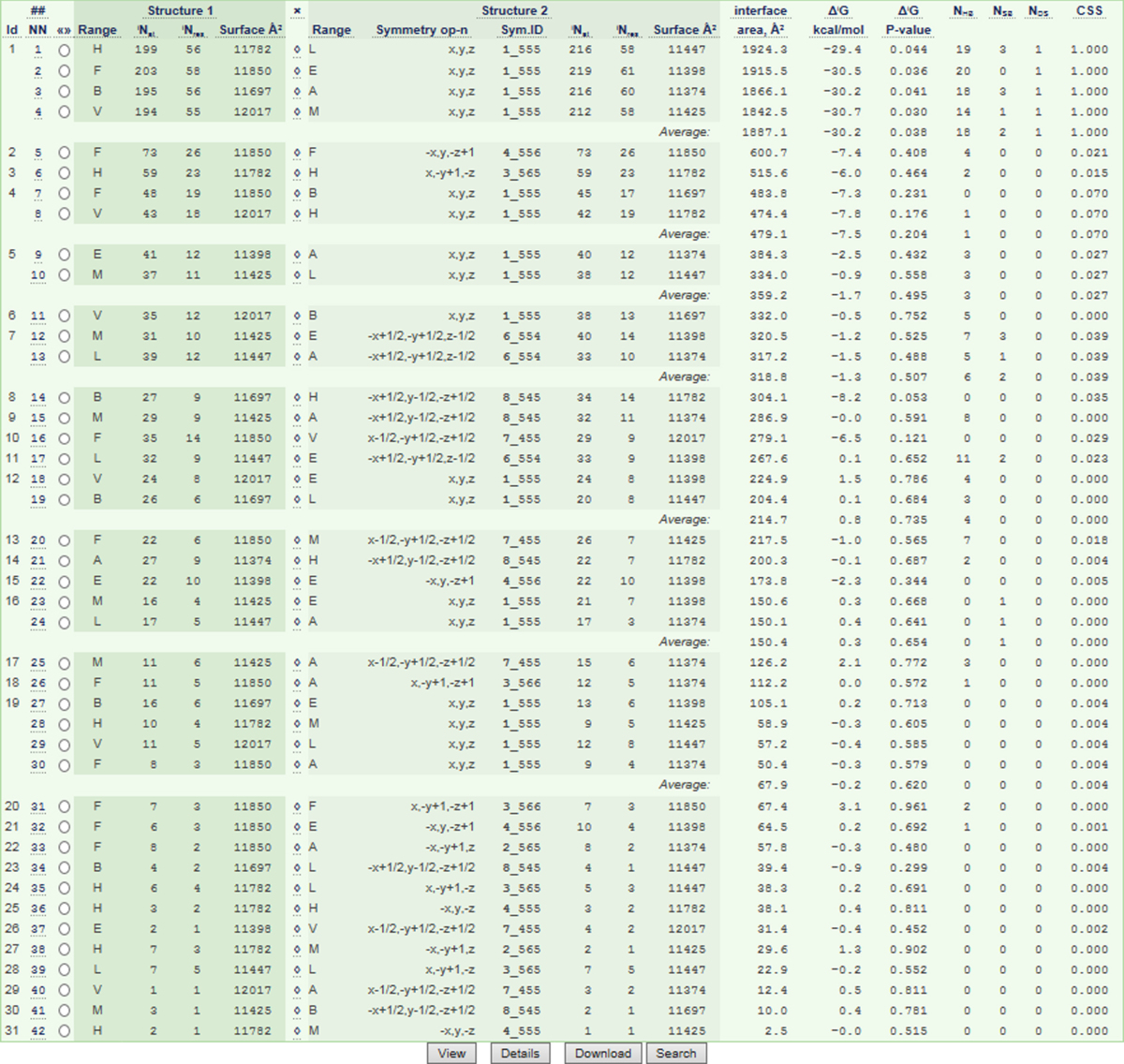

**Fig. 4:**
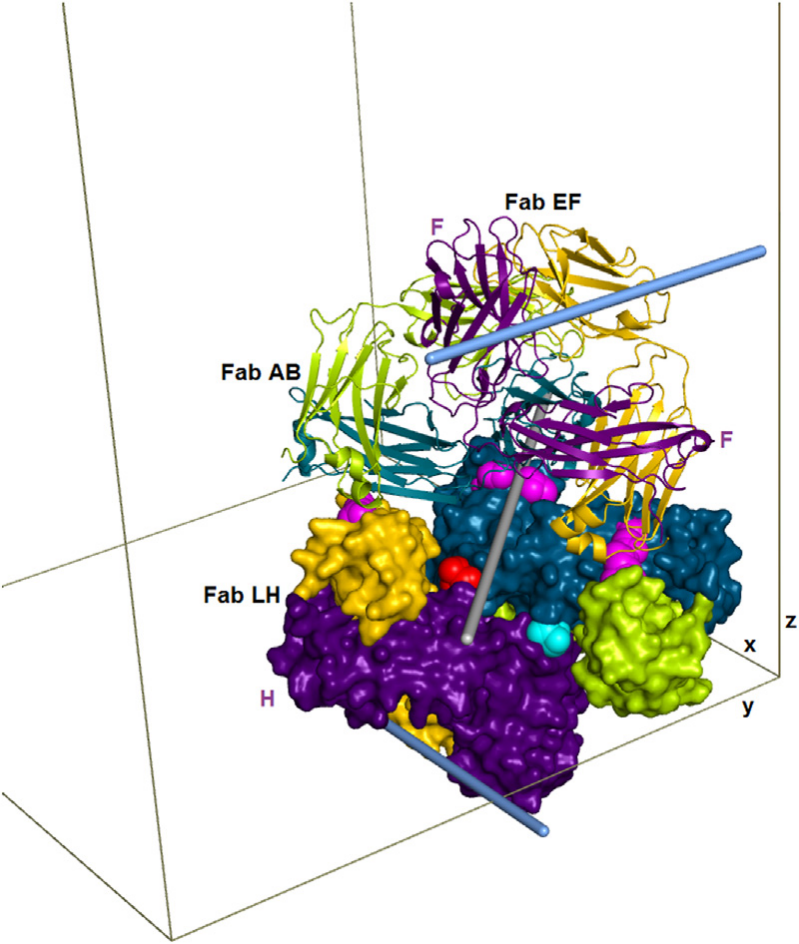
Asymmetric unit of 5K8A. The dimer in Fig. 3 (Fabs LH and MV) is redrawn here using all surfaces, keeping the red and cyan residues at the dimer interface. Above that dimer, ribbons are used to show the other two Fabs, AB and EF, using the same colors, with dark colors for all heavy chains. Three magenta patches indicate the 3 interfaces where the upper dimer touches the lower (corresponding to rows with Id=6 and 12 in Table 2). The gray cylinder indicates the pseudodyad that runs diagonally thru the center of the asymmetric unit. This pseudodyad corresponds to the twin operator K,H,-L. The upper and lower dimers superimpose with root-mean-square deviation (C-alpha atoms) of 0.0977 nm. The blue cylinders show crystallographic dyads where the F and H chains form key interfaces that are shown in Fig. 5.

**Fig. 5:**
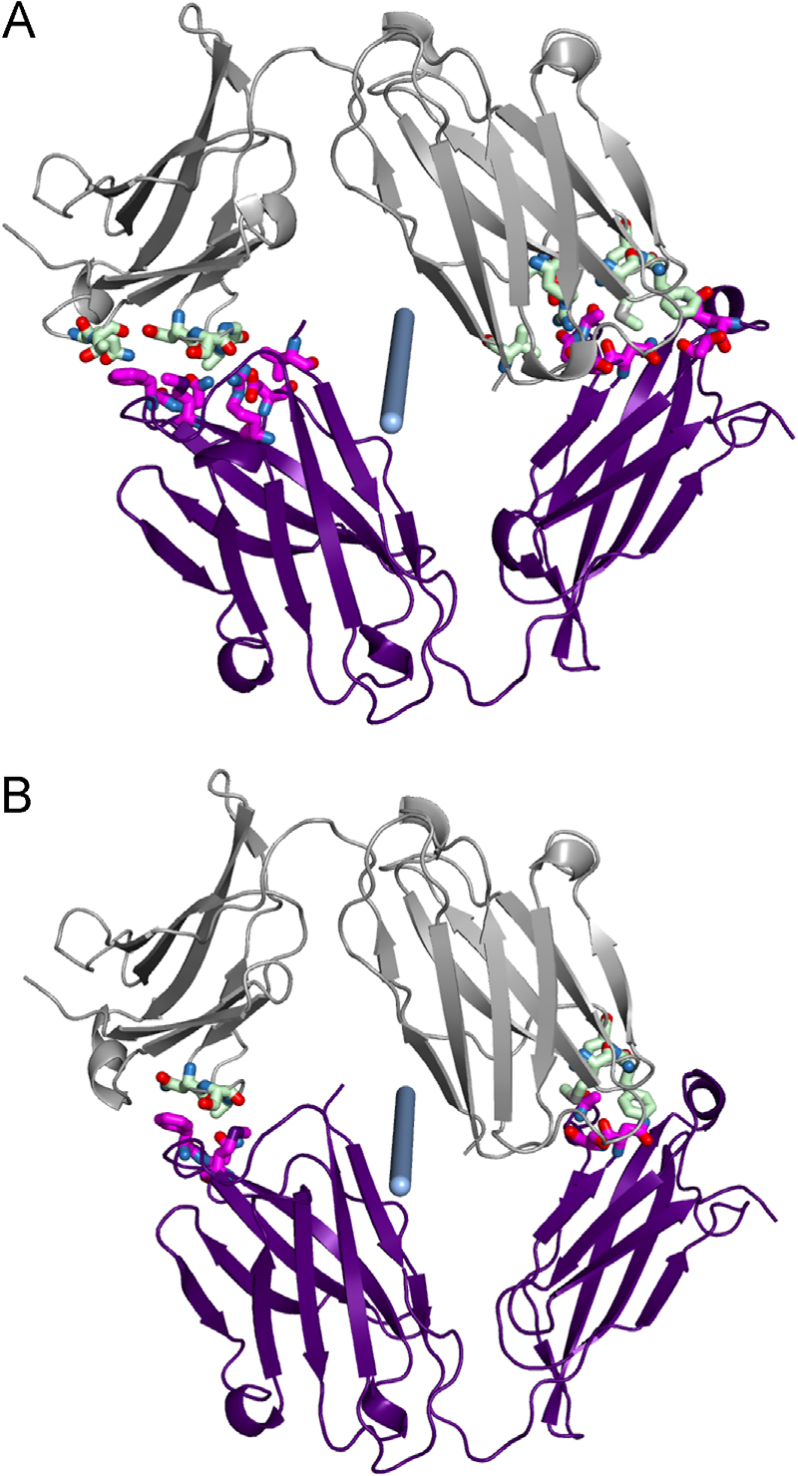
Pseudosymmetry-related interfaces for the F chain (5a) and H chain (5b). Each interface is itself perfectly dyadic; the blue cylinders are the same as in Fig. 4. The crystallographic dyads lie along the y and x directions. These interfaces correspond to Table 2 rows Id=2 and Id=3, where areas and energies are listed. Note that the two interfaces are similar but that a shift of about 0.3 nm makes the F chain interface somewhat larger than for the H chain interface.

The structure contains four independent but similar copies of the complete Fab fragment. Pairwise root-mean-square deviations (RMSD, over C-alpha atoms) among the four Fabs range between 0.05 nm and 0.09 nm. Fig. 2 shows the similarity among the four. Further analysis of the protein and its structure is given in [1]. PDB searches over the most similar unit cells show that the crystal packing in 5K8A is unique. There are 18 other PDB structures with both the a and b axes between 14.4 nm and 15.4 nm, the c axis between 18.8 nm and 20.2 nm, and cell angles between 89 and 91 degrees; none of these 18 are Fabs. Table 2 lists in order of area the contact interfaces among the 8 crystallographically unique chains (four light and four heavy) in the crystal. The first 4 listed contacts, with areas of about 20 nm^2^ (the PISA-provided Table uses units of Å^2^, so the listed area values are about 2000) are the biological light-heavy interfaces within the 4 Fab molecules designated LH, MV, AB and EF. The overall packing of the 4 Fabs is shown in Fig. 3, Fig. 4. Fig. 3, Fig. 4, Fig. 5, along with RMSD calculations, were made using the PyMOL molecular graphics system version 1.5.0.4 (Schrodinger, LLC).

After the large biological interfaces in Table 2, listed with Id = 1, the rest of the Table gives data for the crystal packing interfaces. The first two of these (rows with Id = 2 and Id = 3) are a pseudosymmetric pair, roughly similar but differing by about 0.3 nm and differing in their specific residue interactions, as shown in Fig. 5. The next four listed contacts form the LH+MV 'dimer of Fabs' shown in Fig. 3, and its pseudosymmetric partner AB+EF shown in Fig. 4. The next contact (Id = 6) along with contact 12, joins the dimers to form the whole asymmetric unit (Fig. 4). Thus the largest contacts produce vertical columns of successive asymmetric units. These are known as periodic bond chains (PBC's). These PBC's contain both the Id = 2 and Id = 3 interfaces in alternation and have pseudo-fourfold helical symmetry. Although the calculated energies (Table 2) of these similar interfaces imply that contact 2 (Fig. 5a) is energetically preferred, its repetition (without contact 3) distorts the PBC and produces a nonintegral, underwound helix (not shown) incompatible with a regular lattice. This provides an explanation for the observed pseudosymmetry: it weakens the PBC, but converts its helical pitch to an integer value (n = 4), thus enabling a regular lattice. This case of pseudomerohedral twinning reflects a mixture within the crystal of domains in which these pseudosymmetric substructures exchange places by the observed rotation K, H, -L, which corresponds to the peudodyad in Fig. 4.

## 2 Materials and methods

The Fab fragment was produced from the NIST mAb as described [1] and prepared for crystallization by buffer exchange into 20 mmol/L histidine, pH 6.0 and concentration to 25 mg/mL. Crystal screening against about 500 conditions yielded clusters of rectangular bars (crystal form 1) from several low-pH conditions that were optimized to 12% polyethylene glycol 4000, 25 mmol/L magnesium chloride, 40 mmol/L ammonium sulfate, 80 mmol/L sodium citrate, pH 3.4. As the best form observed during initial screening, these crystals were extensively cultivated. They diffracted to 0.27 nm with orthorhombic cell 9.6 nm, 13.6 nm, 17.8 nm, but due to problems with the crystals (highly compound and thin) and diffraction (anisotropic disorder), they appeared unsuitable for structure determination, and further screening was undertaken. This resulted in conditions for form 2, optimized to 1.9 mol/L ammonium sulfate, 200 mmol/L lithium sulfate, 100 mmol/L histidine, pH 7.0. Crystals of form 2 appeared initially as birefringent spheres, growing into ellipsoids (Fig. 1), then gradually expressing edges but remaining somewhat rounded as they reached a 150 μm maximum diameter. A crystal with length 100 μm was dipped for 2 seconds in 20% glycerol solution, frozen by immersion in liquid nitrogen, and then kept at cryogenic temperature through data collection. A 99.7% complete diffraction dataset to 0.20 nm resolution was collected at beamline 23-ID of the Advanced Photon Source at Argonne National Labs, using an ADSC Q315r detector (ADSC, Poway, CA). Using the program HKL3000 (HKL Research, Inc, Charlottesville, VA), data were initially reduced in space group I422 but subsequently in I222 (see below), resulting in the statistics in Table 1.

Diffraction data were phased by molecular replacement using the program PHASER to locate all four Fabs in the asymmetric unit [5]. The probe was made by removing all ligands and CDR loops from precedent PDB structure 3QWO. Refinement utilized the program Refmac 5.8.0135 within the CCP4i suite [6]. Initial refinements failed to reduce R factor values, and led to very weak maps, leading to testing several tetragonal and orthorhombic space groups, along with tests for possible twinning. Success followed data reduction in space group I222, combined with Refmac's twin refinement feature, which inferred a twinning operator of K, H, -L (corresponding to a rotation of 180° around the xy diagonal) and a twin fraction of 0.6. Maps were then used to build the structure in the conserved and core regions; then most external and loop regions, and finally the CDR loops. Hinges and water molecules were added using unbiased difference maps as guides. The final rms bond length deviation from ideal values is 0.008 nm, and the free-R value is 0.24. The structure and further experimental statistics have been deposited as 5K8A in the Protein Data Bank.
