# Supplementary material for: Data on crystal organization in the structure of the Fab fragment from the NIST reference antibody, RM 8671
Source: Data Brief. 2017 Nov 8;16:29–36. doi: 10.1016/j.dib.2017.11.013 (PMC5686461; doi:10.1016/j.dib.2017.11.013)
Supplement: Supplementary file 1 — Transparency document [file mmc1.docx]

Conflicts of Interest: NONE
